# Exploring Fungal Diversity in Seagrass Ecosystems for Pharmaceutical and Ecological Insights

**DOI:** 10.3390/jof10090627

**Published:** 2024-09-02

**Authors:** Oshadi Rajakaruna, Nalin N. Wijayawardene, Susantha Udagedara, Prabath K. Jayasinghe, Sudheera S. Gunasekara, Nattawut Boonyuen, Thushara C. Bamunuarachchige, Kahandawa G. S. U. Ariyawansa

**Affiliations:** 1 Centre for Yunnan Plateau Biological Resources Protection and Utilization, College of Biological Resource and Food Engineering, Qujing Normal University, Qujing 655011, China; koshadirajakaruna@gmail.com (O.R.); nalinwijayawardene@yahoo.com (N.N.W.); 2 Department of Plant Sciences, Faculty of Science, University of Colombo, Colombo 00300, Sri Lanka; 3 Tropical Microbiology Research Foundation, Pannipitiya 10230, Sri Lanka; 4 Blue Resources Trust, Colombo 00700, Sri Lanka; susantha@blueresources.org; 5 National Aquatic Resources Research and Development Agency (NARA), Crow Island, Colombo 01500, Sri Lanka; prabathj@nara.ac.lk (P.K.J.); sudheera@nara.ac.lk (S.S.G.); 6 National Center for Genetic Engineering and Biotechnology (BIOTEC), National Science and Technology Development Agency (NSTDA), 113 Thailand Science Park, Phahonyothin Road, Khlong Nueng, Khlong Luang, Pathum Thani 12120, Thailand; nattawut@biotec.or.th; 7 Department of Bioprocess Technology, Faculty of Technology, Rajarata University of Sri Lanka, Mihintale 50300, Sri Lanka

**Keywords:** biogeography, endophytes, epiphytes, marine ecosystems, pathogens, species diversity

## Abstract

Marine ecosystems are important in discovering novel fungi with interesting metabolites that have shown great potential in pharmaceutical and biotechnological industries. Seagrasses, the sole submerged marine angiosperm, host diverse fungal taxa with mostly unknown metabolic capabilities. They are considered to be one of the least studied marine fungal habitats in the world. This review gathers and analyzes data from studies related to seagrasses-associated fungi, including taxonomy and biogeography, and highlights existing research gaps. The significance of the seagrass–fungal associations remains largely unknown, and current understanding of fungal diversity is limited to specific geographical regions such as the Tropical Atlantic, Mediterranean, and Indo-Pacific. Our survey yielded 29 culture-dependent studies on seagrass-associated endophytic and epiphytic fungi, and 13 miscellaneous studies, as well as 11 meta-studies, with no pathogenic true fungi described. There is a significant opportunity to expand existing studies and conduct multidisciplinary research into novel species and their potential applications, especially from understudied geographical locations. Future research should prioritize high-throughput sequencing and mycobiome studies, utilizing both culture-dependent and -independent approaches to effectively identify novel seagrass-associated fungal taxa.

## 1. Introduction

Fungi are a diverse group of organisms inhabiting a wide range of environments, playing significant functions in challenging ecosystems [1,2]. They can range from unicellular to filamentous taxa and exhibit various life modes, such as pathogens, saprobes, endophytes, and epiphytes [3]. All these different life modes of fungi contribute to the overall functioning of an ecosystem, though some may not always be beneficial, such as phytopathogenic fungi [4] and clinically important fungi [5]. Approximately, 156,000 species have been scientifically documented in Species Fungorum 2024 [6]. The global richness of fungi has been a popular topic among mycologists with diverse predictions made over the past three decades using various approaches [7,8,9,10,11,12]. Hawksworth and Lücking [10] estimated there to be between 2.2 to 3.8 million fungal species, but the most recent estimate by Niskanen et al. [11] predicts 2 to 3 million species to be more realistic, with over 90% of these species yet to be revealed. Marine fungi have been identified as a potential group for bridging gaps in missing species [11,13] and for discovering novel biological substances [14].

Microorganisms are considered ubiquitous in marine ecosystems, despite the environmental limitations posed by high salinity, low temperature, low water availability, and oligotrophic conditions [15]. Microorganisms represent 90% of the total oceanic biomass [16], making marine ecosystems a potential habitat for discovering novel microorganisms. This largely understudied habitat is rich in microorganisms producing bioactive natural products [17]. Marine fungi are one of the important groups of fungi that mycologists have continuously studied for over 50 years [18,19]. According to www.marinefungi.org (accession on 14 July 2024), 2041 species have been reported as marine fungi [19]. According to Calabon et al. [20] and Devadatha et al. [21], the majority of these taxa have been documented from mangroves and salt marshes. Additionally, substrates such as algae, silt, driftwood, and seagrasses have been researched globally [22]. While over 10,000 fungal species from different marine habitats have been predicted, less than 20% of them have been described [19]. Wijayawardene et al. [23] emphasized the need for investigating marine fungi from various microhabitats in understudied geographical regions and highlighted the role of multidisciplinary sciences in discovering novel fungi and understanding their potential uses.

For over 150 years, it has been known that plants and microorganisms have intimate relationships that affect overall plant fitness, growth, and survival [24]. Consequently, studying plant–microbe associations to uncover the elements of their interactions has become a topic of interest in modern times. As a component of the marine ecosystem, seagrasses host a diverse community of microorganisms, including bacteria, archaea, fungi, microalgae, and viruses [16]. These microbes have a fundamental impact on the physiology and well-being of seagrasses while playing a major role in controlling biogeochemical processes within entire seagrass meadows [16].

During our ongoing study on seagrass-associated fungi in the Puttalam lagoon, Sri Lanka, we isolated over 40 different fungal species from two seagrass species, namely, *Enhalus acaroides* (Linnaeus f.) Royle, 1839 and *Oceana serrulata* (R.Brown) Byng & Christenh. Concurrently, our literature survey revealed that a considerable number of studies on seagrass fungi (including fungus-like taxa) have been carried out, but the data (taxonomy, classification, applications, biogeography, and ecological data) are scattered. This review and opinion paper aims to compile the available data on seagrass-associated fungi (focusing on taxonomy, biogeography, and industrial applications) with the collaboration of fungal taxonomists, marine ecologists, and industry experts. Furthermore, we intend to highlight research gaps and emphasize the need for more research on seagrass-associated fungi to expand future studies in this field.

## 2. Marine Fungi

‘Marine fungi’ are defined in multiple ways in the literature. One of the earliest definitions is based on their requirement to grow in saline water [25]. The most quoted definition provided by Kohlmeyer and Kohlmeyer [26] restricts marine fungi to two ecological groups: obligate and facultative marine fungi. Obligate marine fungi exclusively grow and sporulate in marine environments, while facultative species sporulate in marine environments but can grow in freshwater or terrestrial environments [26,27]. Later, a broader definition was provided by Pang et al. [28], where marine fungi were defined as fungi that are repeatedly recovered from marine habitats and either: (1) grow and/or sporulate on substrates in marine environments; (2) form symbiotic relationships with other marine organisms; or (3) adapt and evolve at the genetic level or are metabolically active in marine environments. Recently, Pasqualetti et al. [15] defined fungi obtained from marine environments as “marine-derived” as they are isolated from marine sources. This definition includes obligates, facultatives, and fungi arising from dormant terrestrial or freshwater propagules. To address the confusion regarding the applicability of these terms, a revision of the taxonomy and a deeper understanding of the metabolomic basis of marine fungal life are necessary.

In general, marine fungi have been recovered from different habitats, including mangrove plants, macroalgae, coral reefs, drift and submerged wood, sponges, sea ice, sea garbage, seagrasses, deep-sea and benthic sediments, hydrothermal vents, oxygen-deficient environments, and the water column [29,30,31]. To provide a structured reference for marine fungal habitats, Wijayawardene et al. [23] defined these habitats as ‘coastal terrestrial ecosystems’, ‘semi-coastal marine ecosystems’, ‘coastal marine ecosystems’, and ‘deep marine ecosystems’. Despite this categorization, fungal associations in many marine habitats, especially seagrasses, remain understudied, which is the primary focus of this review.

## 3. Seagrasses

Seagrasses are flowering plants (angiosperms) found in shallow marine waters [32] and are adapted for fully submerged conditions [33]. They can exist as monospecific or multispecific meadows, often extending over a larger surface area covering the seabed [34].

Despite their crucial role as foundation species in coastal ecosystems [35], seagrass populations are declining at an alarming rate. Studies indicate that the extent of seagrass beds has been declining by 110 km^2^ per year since 1980, with 29% of their total extent lost since seagrass regions were first recorded in 1879 [36]. Consequently, there is an urgent necessity for focused worldwide conservation initiatives to protect the existing seagrass meadows [37].

### Seagrass Distribution and Diversity

Seagrasses are widely distributed along tropical and temperate shallow coastal waters spanning 159 countries on six continents, making them one of the most widespread coastal habitats on Earth [38,39]. They are often associated with important marine habitats such as corals, bivalve reefs, and mangroves in tropics and marshes, and kelp forests in temperate regions [40].

According to the estimates from the UNEP World Conservation Monitoring Centre (WCMC), the spatial distribution of seagrasses was approximated at 177,000 km^2^ in 2001 [41]. However, a recent study by McKenzie et al. [42] revised this estimate, indicating a global seagrass distribution of 160,387 km^2^, with a high to moderate level of confidence. Furthermore, UNEP [39] highlighted that the estimated global extent of seagrass ranges between 300,000 and 600,000 km^2^. The depth limits of seagrasses are estimated to range from the intertidal zone to 90 m below mean sea level [43].

Seagrasses are currently divided into six families: *Zosteraceae*, *Hydrocharitaceae*, *Posidoniaceae*, *Cymodoceaceae*, *Ruppiaceae*, and *Zannichelliaceae* [44]. These six families contain twelve genera and 72 species worldwide [44]. The extinction risk assessment of the world’s seagrass species reveals that three species are Endangered, seven Vulnerable, five Near Threatened, 48 are categorized as Least Concern, and nine species are Data-Deficient [44]. According to Rasheed and Unsworth [45], nearly 10% of all coastal seabeds are covered with seagrass and they exhibit low taxonomic diversity.

Amongst these, the tropical Indo-Pacific represents one of the most highly diverse seagrass bioregions in the world, accounting for around 35% of the total species. However, this region also faces significant data scarcity, particularly for population spatial distribution data, accounting for around 24% of the data deficiency [38].

## 4. Seagrass-Associated Fungi

Compared to the extensive studies on seagrass ecology, less is known about the diversity and ecological roles played by the fungal communities associated with seagrasses [35]. The fungal community associated with land plants plays a crucial role in influencing plant health and survival. Similarly, in seagrass ecosystems, the fungal community can significantly impact overall seagrass functioning. Understanding these associations can be a valuable tool for future seagrass restoration and conservation projects [46,47].

### 4.1. Seagrass-Associated Endophytic Fungi

In this section, our focus is on seagrass endophytic fungi recovered through culture-dependent approaches. Fungi derived from culture-independent studies will be discussed in the section on seagrass mycobiome studies.

The term ‘endophyte’ (endon = within, phyte = plant) is used to define any organism found within living plant tissues. In mycology, this term often specifically refers to fungi that reside within (inside) the plant tissues for at least part of their lifecycle without causing apparent harm or disease symptoms to the host [48]. The association between endophytes and their host plants can range from “mutualistic to opportunistically pathogenic” in nature [49]. According to Schulz and Boyle [50], endophytes are considered as virulent pathogens when they produce enzymes that damage host tissues to aid in colonization. However, a clear distinction between a pathogenic and non-pathogenic nature is often challenging. Recently, Hardoim et al. [51] suggested that the term endophyte should include all microorganisms that colonize internal plant tissues for all or part of their lifetime. Nevertheless, the distinction between a pathogen and an endophyte becomes apparent when they engage with the plant’s defence system. A pathogen overcomes the plants’ defences, inflicting damage on the host, whereas endophytes overcome plant defences by masking themselves without causing apparent damage or symptoms.

In general, endophytic fungi are considered to enhance the hosts’ ability to tolerate environmental stresses, improve vigour, recycle nutrients, decrease susceptibility to pathogens and pests, and regulate the synthesis of phytohormones and metabolites [52,53,54,55]. In return, host plants provide them with organic nutrients, protection, and assurance of survival [56]. A recent study showed that two endophytic fungi, *Trichoderma* sp. and *Diaporthe* sp., isolated from a seagrass species, *Thalassia testudinum,* demonstrated significant bioactivity against pathogenic *Labyrinthula* infections, which have previously led to extensive seagrass die-offs in many parts of the world [57].

To the best of our knowledge, the first record of intercellular fungi in seagrass was reported by Kuo [58] in 1984, in the leaves of *Zostera muelleri*. Although they did not specifically use the term “endophytic”, the symbiotic and intercellular nature of fungal filaments was demonstrated using microscopy. The first direct evidence of endophytic fungi isolated from living, healthy seagrass tissues was reported by Wilson [59] through the screening of the leaf tissues of *Thalassia testudinum*, *Halodule bennudensis,* and *Syringodium filiforme*. Since then, numerous investigations on the isolation and identification of seagrass endophytic fungi have been published, and all culture-based studies as well as isolated taxa reported thus far are summarized in Table S1.

**Figure 1:**
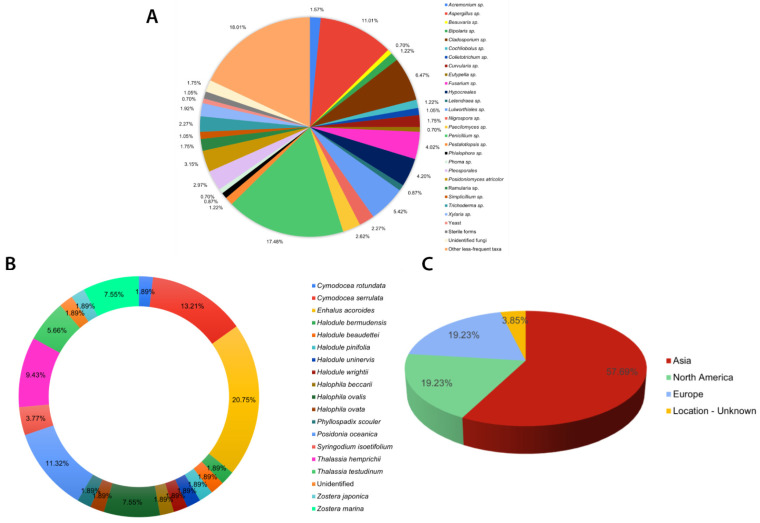
Summary of culture-dependent seagrass endophytic fungal studies listed in Table S1: (**A**) composition of endophytic fungi from different seagrass studies; (**B**) percentage number of studies carried out for each seagrass species; (**C**) continent-wide distribution of sampling sites for seagrass fungal endophytes in the world.

Eurotialean fungi, specifically *Aspergillus* and *Penicillium,* are reported as the dominant endophytic taxa in most culture-dependent studies [60,61,62,63,64] (see Table S1). These two genera, *Penicillium* (100/572) and *Aspergillus* (63/572), account for more than 25% of the listed taxa (Figure 1A). Nevertheless, in most of these publications, fungal identification is restricted only to the generic level without support from DNA sequencing. Therefore, the taxonomic placements may not be very accurate.

Most of these dominant endophytic genera are reported to have a terrestrial origin [60,65,66,67]. While many studies report the absence of obligate marine fungi [66,67,68], some studies such as Cuomo et al. [69], Abdel-Wahab et al. [70], and Mata and Cebrián [61] mention the presence of obligate fungal species. According to Abdel-Wahab et al. [70], employing rigorous surface sterilization during endophytic isolations enhances the recovery of marine-derived fungal species, whereas a direct microscopic examination tends to identify obligate marine fungi. However, the validity of this statement is rather perplexing as the marine-derived, or obligatory, nature cannot be determined by morphological characters alone. In many studies, sterile forms are reported [61,63,66,67] and remain unidentified when morphological methods are used for identifications. In our ongoing study, over 15 endophytic fungi (out of a total of 40) isolated from two seagrass species, *E. acaroides* and *O. serrulata*, lacked any fruiting structures, necessitating molecular identification (Rajakaruna et al., unpublished). Gnavi et al. [71] highlight the need for molecular analyses to properly identify these sterile forms.

Seagrasses are reported to have a low diversity and density of fungal colonization compared to terrestrial plants [60,62,67,68]. However, Shoemaker and Wyllie-Echeverria [72] noted that the number of taxa isolated is “roughly similar” to that of land plants. While these comments appear contradictory, it is important to consider the diversity indices utilized by these authors, the climatic zones of the plants used for comparisons, and the total number of segments screened, before coming to a meaningful conclusion. Moreover, all the above predictions were based on culture-dependent techniques.

Nevertheless, it is stipulated that the low frequency of fungal colonization in seagrasses is attributed to multiple complex interactions between intrinsic and extrinsic factors. The endophytic fungal community in seagrasses is mainly influenced by intrinsic factors including tissue type (“district specificity”) [73], age [60,62], morphological characters [59,60], and the phytochemical composition of tissues [62,67]. The antifungal metabolites produced by seagrasses are reported to limit internal fungal colonization [74]. Additionally, other groups of microbes associated with seagrasses, which produce antifungal compounds, can further reduce endophytic colonization [75].

External factors such as the nutrient content in the water column, water temperature, wind and wave actions, seasonal variations [59,60,61], and other physicochemical factors at the sampling sites may affect the fungal colonization within seagrasses. A recent study by Solé et al. [76] showed that human-generated noise significantly impacts the degradation of fungal symbionts in *Posidonia oceanica* roots, subsequently affecting the normal root functions. Moreover, the absence of mycorrhizal associations in seagrasses is said to be associated with limitations posed by high salinity and oligotrophic conditions in the marine sediments [77]. Although seagrasses are adapted for nutrient uptake through leaves, having root–fungal associations (similar to mycorrhizal associations in land plants) could be beneficial for absorbing nutrients from recalcitrant material under oligotrophic conditions [78]. A summary of these symbiotic root–fungal associations is given in Section 4.1.1.

**Figure 2:**
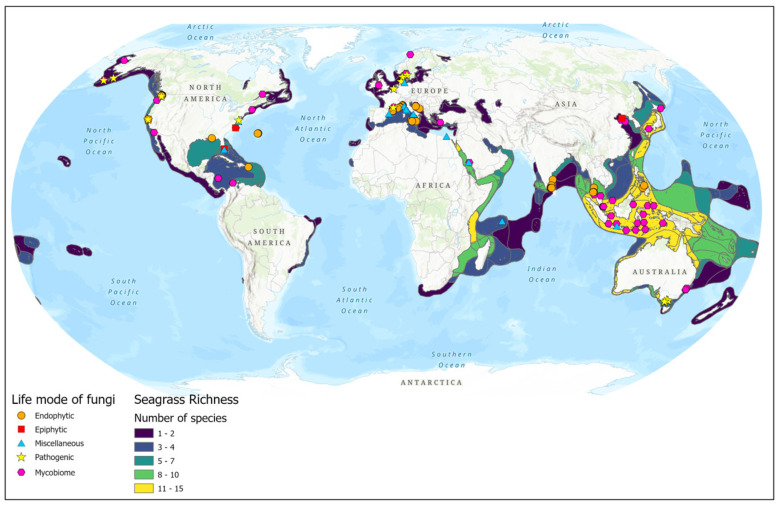
Biogeographic distribution of seagrass fungal studies (based on the data from Tables S1–S4).

Most of the seagrass endophytic fungal research has been conducted in the Asian, European, and North American regions (Figure 2). More than half of the sampling sites are concentrated on the Asian continent (Figure 1C and Figure 2). The majority of studies are confined to a few seagrass species: *Enhalus acaroides*, *Cymodocea serrulata, Posidonia oceanica, Thalassia hemprichii,* and *Zostera* spp. (Figure 1B).

#### 4.1.1. Root-Associated Endophytic Fungi

Some publications have explicitly investigated and uncovered interesting root–fungal relationships in seagrasses. Kuo et al. [79] reported the occurrence of fungi in the peripheral root tissues of two Australian seagrass species, *Posidonia australis* and *P. sinuosa*. To the best of our understanding, this is the first published investigation that specifically addresses the fungal inhabitants in seagrass roots.

Thereafter, for more than a decade, the topic remained unstudied. Nielsen et al. [77] attempted to find arbuscular mycorrhizal (AM) associations in the roots of *Zostera marina* and *Thalassia testudinum*. Since the majority of vascular plants contain these symbiotic associations, the authors aimed to address this gap in seagrass research. However, they were unable to observe AM associations in either seagrass species. The observed *Zostera marina* tissues showed unidentified fungal colonization that was not characteristic of AM. As they assumed, the lack of information on seagrass-associated root mycorrhizae in the 1900s is due to the inherent challenge of publishing negative results.

Torta et al. [80] conducted a study on root mycobiota of *Posidonia oceanica* and obtained a single species which they named *Lulwoana* sp. Without any supportive evidence, they claimed this species was a dark septate endophyte (DSE). Vohník and colleagues conducted numerous investigations to study fungi associated with seagrass roots but found no structures resembling mycorrhiza. However, in their study, Vohník et al. [81] described the presence of dark septate endophytes (DSE) with all typical structures, including extraradical and intraradical dark septate hyphae, dense melanized parenchymatous nets/hyphal sheaths on the root surface, and melanized intracellular microsclerotia for the first time in seagrasses. Subsequently, Vohník et al. [82] isolated three fungal species from the roots of the seagrass *Posidonia oceanica* with the dominant fungal species exhibiting characteristics of DSE. Sequencing data showed that this species belongs to the order *Pleosporales,* representing a new member in the *Aigialaceae*. A similar observation showing narrow endophytic diversity and *Pleosporales* dominance was reported from a culture-independent approach [83]. The mycobiont responsible for these dominant DSE associations in *Posidonia oceanica* was later described as *Posidoniomyces atricolor* [84].

However, the results of Vohník et al. [84] conflicted with the findings of Torta et al. [80] regarding the dominant DSE fungal groups, form, and distribution of fungal colonization. To resolve this, a comprehensive study was later conducted by Vohník et al. [78], at the same localities previously investigated by Torta et al. [80] and the dominant DSE associations in *Posidonia oceanica* was confirmed again as *Posidoniomyces atricolor* and not *Lulwoana* sp., as described by Torta et al. [80] previously. Lefebvre et al. [85] claim the colonization of *Posidoniomyces atricolor* in the degrading tissue of *Posidonia oceanica,* showing its saprobic nature, which extends beyond a typical plant endophytic association. Overall, the findings of these studies indicate that roots of seagrasses are colonized by DSE, but, until now, as already foreseen, no mycorrhizal association has been described in the roots of seagrasses.

In addition to these reports, Marina Carrasco-Acosta recently presented the isolation of an obligate marine fungus, *Cumulospora marina*, from the root tissues of *Cymodocea nodosa* at an IUMBM conference on extremophilic fungi [86]. Furthermore, Wang et al. [87] identified a novel lulworthioid fungus, *Halophilomyces hongkongensis,* colonizing the roots and rhizomes of the seagrass *Halophila ovalis*. However, the contribution of these fungal species on seagrass health is still unknown.

### 4.2. Seagrass Epiphytic Fungi

Epiphytes are spatially different from endophytes and are defined as organisms that live upon the plant surfaces [32]. Similar to endophytes, seagrasses provide excellent habitats for the epiphytic organisms [32,88]. The physicochemical changes in the surrounding water column provide environmental variability, while the host characteristics (such as leaf area) create structural variability for a variety of epiphytic organisms to colonize seagrass surfaces [89]. These variations shape the composition of the epiphytic community and their interactions with the host.

The epiphytic community of seagrasses is primarily composed of algae dominated by the *Rhodophyta*, while fungi remain largely unstudied [90]. In a previous study, fungi were recorded as “rarely found” in the epiphytic community of *Posidonia oceanica* leaves [91]. However, recent studies indicate that epiphytic fungi are abundant in seagrasses, and their true diversity and potential are yet to be fully discovered.

Typically, endophytic fungal isolates are obtained following surface sterilization with sodium hypochlorite and ethanol, whereas epiphytes are isolated using less stringent surface sterilization methods [49]. In several studies, surface sterilization methods are not explicitly specified. Thus, it is reasonable to believe that these studies have isolated both endophytic and epiphytic fungi. Table S2 provides a summary of these miscellaneous forms reported from seagrasses worldwide.

Genera such as *Cladosporium*, *Colletotrichum*, and *Penicillium*, and the order *Hypocreales*, which are often recorded as endophytes, have also been reported as epiphytes in a few studies [92,93]. However, the possibility of contamination, deficiencies in surface sterilization stringency, misidentification of fungi, and human error should also be considered when making inferences from these outcomes. Recently, a novel epiphytic root–fungus symbiosis has been reported to be associated with the roots of the Indo-Pacific seagrass *Thalassodendron ciliatum* [94].

In comparison, studies on the culture-dependent isolation of seagrass endophytic fungi are more common than those on epiphytic fungi. However, several studies conducted thus far have identified bioactive compounds from epiphytic fungi with potential industrial applications (Table 1). Nonetheless, there exists a notable disparity between these findings and the published records on the initial isolation of these fungi.

### 4.3. Pathogenic Phytophthora and Halophytophthora Species of Seagrasses

Studies indicate that seagrass meadows are gradually thinning and diminishing due to the increased occurrence of diseases caused by pathogenic microorganisms. Seagrasses are infected by four main groups of pathogens: *Labyrinthula*, *Phytophthora*, *Halophytophthora*, and *Phytomyxea* [122,123]. Members of *Labyrinthula* (Labyrinthulids) and *Phytomyxea* (Plasmodiophorids) are classified under the Kingdom *Protista* [124,125] while *Phytophthora* and *Halophytophthora* are in the class *Oomycota*, Kingdom *Straminipila* [126].

Thus far, our literature review has revealed no direct records of any major diseases caused by fungal pathogens in seagrasses. Hu et al. [127] reported the bioactivities of *Aspergillus alabamensis*, a “phytopathogenic fungus” isolated from the seagrass *Enhalus acoroides*. However, it is a speculation, and its pathogenicity to seagrass has not been established.

In contrast, considerable scientific attention has been dedicated to *Labriyrnthula* pathogens responsible for the “seagrass wasting disease”, which causes noticeable, extensive losses in many regions of the world. Little is known about the pathogenicity and disease ecology of other groups. Since the focus of this review is on fungal pathogens, we will only look at *Phytophthora* and *Halophytophthora* pathogens, which are considered as fungus-like Oomycetes.

Oomycetes are behaviourally similar but biologically distinct from other main groups belonging to Kingdom *Fungi* [126]. Historically, they were classified under ‘Phycomycetes’ or “lower fungi” [128]. Considering this early classification, a brief summary of *Phytophthora* and *Halophytophthora* species reported in seagrasses is given in Table S3. Relatively few studies have reported the discovery of pathogenic *Phytophthora* and *Halophytophthora* species in seagrasses. *Phytophthora* and *Halophytophthora* infections have been found to reduce the sexual reproduction of *Zostera marina* by sixfold [119]. Later, Govers et al. [129] demonstrated that treating *Z. marina* seeds with copper sulphate can effectively control *Phytophthora* and *Halophytophthora* infections, highlighting the effectiveness of this method in seed-based restoration projects.

Further, these pathogens are sensitive to annual and seasonal variations, and migratory bird species have been shown to impact disease dissemination [130]. Regardless of the significance of these pathogens in seagrass health, many studies are confined to *Zostera* spp. in the northern hemisphere. Comparable to *Zostera* spp., very little is known about the disease occurrence and pathogenicity of *Phytophthora* and *Halophytophthora* in other seagrass species.

### 4.4. Seagrass Mycobiome Studies

Conventional methods for investigating plant-associated fungi involve isolating and cultivating them on artificial media. Many investigations of seagrass-associated fungi have been undertaken using this standard methodology. However, culture-dependent approaches are often associated with inherent limitations, such as the inability of certain microorganisms to grow on culture media or under specific incubation conditions, as well as the masking of slow-growing microorganisms by fast growers [131]. Thus, these approaches often fail to capture the majority of microbial diversity within environmental samples [132].

As a result, culture-independent, high-throughput molecular methods have gained popularity in recent years to reveal the true diversity and abundance of microorganisms in complex environmental samples. A few meta-studies on seagrasses have been conducted focusing on the seagrass species such as *Zostera marina*, *Z. muelleri*, *Posidonia oceanica*, and *Halophilia* spp. Similar to the culture-dependent approaches, these studies report the dominance of *Eurotiales* fungi such as *Aspergillus* and *Penicillium* belonging to the phylum *Ascomycota*. However, unlike culture-dependent studies, the dominance of fungi belonging to the phylum *Chytridiomycota* is reported in a few studies [35,83,133,134]. This is the only phylum of true fungi that reproduces with zoospores (motile spores). Members of a relatively new order in *Chytridiomycota*, *Lobulomycetales*, have been reported in a few studies [35,83,133,134]. A concise summary of mycobiome studies reported thus far is given in Table S4. Designing new primer pairs and blocking oligonucleotides for fungal detection, especially for basal fungal lineages such as *Cryptomycota* and *Chytridiomycota,* can further refine these underrepresented groups associated with seagrasses [135,136].

## 5. Significance of Studying Seagrass-Associated Fungi

The significance of studying seagrass–fungal associations can be addressed from two perspectives. First, understanding seagrass-associated mycobionts can help to protect this vulnerable ecosystem. As previously noted, these relationships can have either a positive or negative impact on the overall health of seagrass beds. However, no studies have been conducted to investigate the molecular and biochemical mechanisms that govern the structure, activity, and function of these communities. A recent study demonstrated that endophytic fungi associated with seagrasses possess the ability to inhibit the growth of the devastative seagrass pathogen *Labyrinthula* spp. [57]. Therefore, comprehending these interactions and monitoring the compositional changes in fungal communities can serve as a crucial tool for seagrass transplant and restoration initiatives [46].

From a human-centric standpoint, seagrass-inhabiting fungi can be a novel reservoir of metabolites, useful in pharmaceutical and various other industrial applications. Since ‘marine drugs’ are becoming an appealing strategy for addressing antimicrobial resistance [95], seagrass-associated fungal communities can be studied for novel bioactive chemicals. However, previous reports on the screening and isolation of metabolites from fungi associated with seagrasses are limited, making it difficult to predict their real potential based on reliable scientific information. Table 1 lists some of the beneficial metabolites of seagrass-associated fungi and their bioactivities reported thus far. These bioactive metabolites are known to have antimicrobial, anticancer, anti-inflammatory, and antiviral properties. Some notable bioactive metabolites identified from seagrass mycoflora include Cladionol A [110], Sansalvamide [109], Malformin A1 [104], and Halovir A [106]. Peterson et al. [137] report the use of an omics-based high-throughput approach, a rather new approach for rapid bioactivity testing for seagrass-associated fungal metabolites.

Moreover, several publications report the production of lignocellulosic enzymes [114,115], xylan-degrading enzymes [116], and chitin-modifying enzymes [63] from seagrass-associated fungi. These enzymes have a wide range of biotechnological applications. For example, lignocellulosic and xylan-degrading enzymes are important in the biofuel industry, textile industry, paper and pulp industries, and in bioremediation [114,116]. However, to the best of our knowledge, no enzyme derived from a seagrass-associated fungus has reached mass-scale industrial production. This underscores the need for future studies in upscaling production following successful screening assays. Recently, a few studies have highlighted the potential of seagrass-associated fungi in agriculture, particularly as biocontrol agents and alleviating stress responses such as salinity stress [120,121,127].

## 6. Future Prospects

Currently, there is an emphasis on discovering new taxa from unexplored geographic regions that are quite promising and multifaceted. Nevertheless, it is essential that we incorporate the advances in mycology to expand fundamental studies (e.g., taxonomy based on polyphasic approaches and sequence-based nomenclature to name the Dark taxa). Hence, we recognize several key aspects to continue the research of fungi associated with seagrasses: 1. biodiversity exploration; 2. ecological role understanding; 3. biotechnological potential; and 4. use of emerging technologies.

### 6.1. Biodiversity Exploration

The exploration of biodiversity in the context of seagrass-associated fungi, particularly in uncharted regions such as parts of Southeast Asia and Northern and Southern America, presents an area rich with potential and complexity [22,46,135,138,139]. Seagrass ecosystems, known as biodiversity hotspots, are often under-researched regarding their fungal communities. These ecosystems may host unique fungal species adapted to specific seagrass environments, offering significant opportunities for discovering new fungal species and genera [80,138,140,141,142].

The geographical diversity of these regions suggests that the fungal diversity associated with seagrass beds could vary significantly, influenced by local environmental factors such as climate, water salinity, and the types of seagrasses present. This diversity is crucial for understanding the ecological roles these fungi play, from nutrient cycling and decomposition to aiding seagrasses in defence against pathogens and environmental stressors [16,93,143].

Advancements in molecular techniques, especially high-throughput DNA sequencing, have revolutionized the identification and cataloguing of fungal species. These methods are more efficient and accurate than traditional ones, capable of identifying even non-culturable fungi and uncovering cryptic species—different species previously thought to be the same due to similar appearances [70,84,133].

Exploring these ecosystems could also reveal new symbiotic relationships between fungi and seagrass or interactions with other microorganisms like bacteria and algae [35,134]. Additionally, understanding how these fungi have adapted to their specific environments can offer insights into their potential responses to global environmental changes, such as rising sea temperatures and ocean acidification [144,145].

Furthermore, each new fungal species discovered contributes to the global understanding of biodiversity, which is vital not just for academic knowledge but also for informing conservation strategies and understanding ecological balances. As seagrass meadows are among the most threatened ecosystems globally, knowing the full range of biodiversity within these areas is essential for their effective management and conservation. Overall, this exploration into the uncharted realms of seagrass-associated fungi holds the key to unlocking the complexities of these ecosystems and significantly contributes to our understanding of marine biodiversity and ecosystem health [22,146].

### 6.2. Ecological Role Understanding

Understanding the ecological roles of fungi in seagrass ecosystems is vital. This includes their roles in nutrient cycling, decomposition processes, and interactions with other microbial communities. As we uncover more about these fungi, we can better understand the overall health and functioning of seagrass ecosystems.

In marine ecology, the ecological roles of fungi within seagrass ecosystems are of paramount importance, encompassing a diverse array of functions that are crucial for the health and functionality of these underwater habitats. Fungi play a vital role in nutrient cycling, breaking down organic matter and releasing essential nutrients back into the environment, thereby maintaining the nutrient balance in these often nutrient-poor marine settings [73,147]. Additionally, they are key players in the decomposition processes, aiding in the breakdown of dead plant materials such as seagrass leaves and roots. This decomposition not only contributes to the recycling of organic matter but also supports the detritus-based food web that is central to the health of the seagrass ecosystem [148,149].

The interactions of fungi with other microbial communities within these ecosystems, including bacteria, viruses, and algae, are also significant. These interactions, which can range from symbiotic to competitive, affect the distribution and abundance of various microbial species and play a crucial role in the microbial dynamics of the ecosystem [150,151]. The secondary metabolites produced by these microbes impact the fungal colonization, which, in turn, can affect the general health of seagrass [75,152,153,154]. Interestingly, Nerva et al. [155] demonstrated the importance of mycoviruses, a group of viruses which affect fungi, in influencing the overall health of *Posidonia oceanica*.

Moreover, the health of seagrasses themselves can be directly influenced by the fungi associated with them. While some fungi provide protective benefits, helping seagrasses withstand environmental stressors or deter pathogens, others might be pathogenic and detrimental to seagrass health [16,147]. Conversely, different antifungal metabolites produced by seagrasses can affect internal fungal colonization [75,156,157].

Furthermore, fungi within seagrass ecosystems can act as bioindicators of environmental changes. Variations in fungal communities can signal alterations in environmental conditions, such as pollution or changes in water temperature and salinity, providing valuable insights into the health and stability of these ecosystems [158,159]. For instance, the Seagrass Microbiome Project launched in 2014 aims to identify seagrass–microbe interactions that can reveal important information about seagrass ecology, evolution, and function [160]. Additionally, the role of fungi in the decomposition process has implications for carbon sequestration in seagrass meadows, a significant factor in global carbon dynamics [161,162].

### 6.3. Biotechnological Potential

The biotechnological potential of marine fungi, particularly those associated with seagrass ecosystems, is a burgeoning field of research with significant implications for various industries, including medicine, agriculture, and industrial processes [163,164,165]. Marine fungi are a largely untapped resource, known for their unique ability to produce novel bioactive compounds [166,167]. These compounds, often not found in terrestrial fungi, arise from the adaptation of fungi to the challenging marine environment, characterized by high salt concentrations, varying pressure conditions, and intense competition for resources [168,169].

In medicine, the unique bioactive compounds derived from marine fungi have shown promise in the development of new pharmaceuticals. These compounds can have diverse biological activities, including antibacterial, antiviral, antifungal, anti-inflammatory, and anticancer properties. For example, some compounds might inhibit the growth of cancer cells or bacteria resistant to current antibiotics, offering new avenues for treatment where traditional medicines are failing [166,170].

In agriculture, these fungi could be a source of new biopesticides or growth enhancers. Given their origin in a highly competitive and harsh environment, these fungi may produce substances that are effective in controlling agricultural pests or diseases, potentially reducing the reliance on synthetic chemicals that can be harmful to the environment [164,171].

The industrial sector could also benefit from enzymes and other molecules produced by seagrass-associated fungi. These enzymes might be particularly useful in processes that require tolerance to saline conditions, such as in certain bioremediation applications or in the processing of marine-derived materials [172,173,174]. As reported by Panno et al. [171], the test fungal isolates recovered from seagrasses exhibit no degradative properties in their enzyme activity in high salt concentrations. This indicates that these enzymes could be valuable for future biotechnological applications that operate under extreme physiochemical conditions. Further, Raghukumar et al. [117] demonstrated the use of a seagrass-derived fungus to remove and detoxify wastewater from molasses-based alcohol distilleries, demonstrating their ability for bioremediation while enhancing the sustainability in biorefinery processes.

Moreover, the exploration of these fungi for biotechnological applications also contributes to the understanding of their ecological roles and potential for sustainable utilization. By identifying and harnessing these bioactive compounds, not only can new, potentially groundbreaking products be developed, but it also encourages the conservation of marine ecosystems like seagrass meadows, which are vital for the health of the marine environment [93,175,176].

### 6.4. Use of Emerging Technologies

The use of emerging technologies such as remote sensing, artificial intelligence (AI), machine learning in ecological studies, and nanotechnology can revolutionize our understanding of seagrass-associated fungi. These advanced technologies offer powerful tools for gaining new insights into the distribution, health, and ecological roles of these fungi in marine ecosystems [177,178,179].

Remote sensing technology, including satellite imaging and aerial photography, can monitor the health and extent of seagrass meadows over large areas and over time. This technology enables scientists to detect changes in seagrass coverage and condition, which can be indicative of the health of the associated fungal communities [180]. For instance, a decline in seagrass health might suggest issues such as disease or environmental stress, potentially linked to changes in fungal communities. Remote sensing also allows for the mapping of seagrass habitats, providing valuable data for conservation and management efforts [181,182].

Artificial intelligence and machine learning are rapidly becoming indispensable in ecological research. These technologies can process and analyze vast amounts of data much faster and more accurately than traditional methods [179]. In the context of seagrass-associated fungi, AI can be used to analyze complex datasets from remote sensing, genetic sequencing, and ecological surveys to identify patterns and relationships that might be invisible to the human eye. For example, machine-learning algorithms can help in predicting the distribution of fungal species based on environmental variables or in identifying changes in fungal communities in response to environmental stressors [183,184].

Furthermore, the integration of AI with genomic studies, such as metagenomics and high-throughput sequencing, is particularly promising. This integration allows for the rapid identification and classification of fungal species, even those that are rare or previously unknown. AI algorithms can analyze genetic data to uncover relationships between fungal species, their adaptation strategies, and their interactions with seagrasses and other marine organisms [185].

The production and utilization of nanoparticles using fungi, which is also known as myconanotechnology, is also an emerging topic of research [186]. These fungal-derived nanoparticles have a low toxicity and are eco-friendly, compared to the conventional nanoparticles synthesized by chemical or physical means [187]. As seagrass-associated fungi produce unique metabolites, they can hold unique biochemical mechanisms to generate nanoparticles with diverse chemical characteristics. Further, silver nanoparticles synthesized by this biological method can simplify the synthesis process by eliminating the extra steps required to prevent particle aggregation [188]. According to Abdelrahman et al. [189], it is possible to screen and optimize seagrass-associated fungi for nanoparticle synthesis, leading to nanomaterials with different bioactive properties.

The application of these emerging technologies in the study of seagrass-associated fungi not only enhances research capabilities but also contributes to more effective conservation strategies. By providing a more comprehensive and nuanced understanding of these ecosystems, technology can help in predicting the impacts of environmental changes and human activities, thereby informing management and restoration efforts [95,133]. More importantly, understanding the significance of fungal genetic resources through these new approaches can be used as a valuable tool for in situ conservation and support seagrass reforestation.

## 7. Conclusions

Seagrass beds are distinct ecosystems that serve as a reservoir for a diverse array of microbes, including fungi. These fungi can engage in different relationships with their host, spanning from mutualistic to potentially pathogenic. Culture-dependent and culture-independent methods are currently employed to study the diversity of seagrass-associated fungi.

The majority of culture-based methods concentrate on seagrass inhabiting endophytic fungi. Epiphytic fungi are often overlooked, or it could be argued that they are not easily identified directly, due to the challenges associated with handling samples during initial isolation. Thus far, *Eurotiales* species such as *Aspergillus* spp. and *Penicillium* spp. dominate the fungal community associated with seagrasses. There have been no reports of mycorrhizal associations in seagrass roots so far, although other distinct fungal associations have been discovered. Infections by *Phytophthora* and *Halophytophthora* have been documented in seagrasses, which were formerly classified as lower fungi. However, diseases caused by true fungi in seagrasses remain largely unidentified.

Overall, the majority of culture-based studies are confined to *Enhalus acaroides*, *Cymodocea serrulata*, *Posidonia oceanica*, *Thalassia hemprichii*, and *Zostera marina*. Meanwhile, mycobiome studies have been carried out on *Zostera* spp., *Halophilia* spp., and *Posidonia oceanica*. With 72 seagrass species worldwide, there is a significant opportunity to expand both culture-dependent and -independent research on a global scale. Regions with rich diversity, such as the Tropical Indo-Pacifics along the east coast of Africa, lack sufficient data.

The harsh environment in marine ecosystems may lead to the accumulation of metabolites in seagrass-associated fungi, which could be employed in extreme physiological conditions. These fungi could also be investigated as a source of new pharmaceutical lead compounds for development. Recently, a few attempts to employ these fungi to alleviate stress responses in plants have been reported. However, there is a lack of studies on their applications, and their full potential is still unknown.

Rapidly disappearing seagrass beds highlight the importance of advancing research to preserve their valuable genetic resources before they are lost without documentation. Exploring the ecological significance and relationships of these seagrass–fungal associations can aid in future seagrass restoration and transplantation initiatives. The application of modern technologies such as omics-based methods and AI can enhance research on seagrass fungi even further.

## Figures and Tables

**Table 1:** Bioactivities of metabolites isolated from seagrass-derived fungi.

Bioactivity	Bioactive Compound	Fungal Taxa with Bioactivity	Host Seagrass Species	Life Mode	Reference
Broad spectrum antimicrobial activity (antifungal and antibacterial activity against the tested isolates)	Not isolated	Not identified	*Zostera marina*	Epiphytic	[95]
Mild antibacterial activity against *S. aureus* ATCC25923 and methicillin-resistant *S. aureus*	Chrysophanol and Emodin	*Bipolaris* sp.	*Halophila ovalis*	Not specifically given (from leaves)	[96]
Antimicrobial activity against *Staphylococcus aureus* and anti-quorum sensing effect against *Pseudomonas aeruginosa*	Not isolated	*Penicillium crustosum*	*Posidonia oceanica*	Not specified	[97]
Mild antibacterial and antifungal activity	Zearalenone	*Fusarium* sp.	*Thalassia hemprichi*	Not specifically given (from leaves)	[98]
Antimicrobial activities (antibacterial and antifungal)	Not isolated	Few active isolates identified	*Cymodocea serrulata*, *Halophila ovalis* and *Thalassia hemprichii*	Endophytic	[65]
Antimicrobial activity against 10 human pathogens	Not isolated	Few active isolates identified	*Enhalus acoroides*	Endophytic (from leaves, roots, and rhizomes)	[62]
Antibacterial activity against *Micrococcus luteus*	Not identified	Fungi not identified	*Halophila ovalis, Thalassia hemprichii.* and *Syringodium isoetifolium*	Endophytic	[99]
Significant antifungal activity against *Penicillium griseofulvum* and *Aspergillus favus*	Terperstacin and 19-acetyl-4-hydroxydictyodiol	*Mariannaea humicola*	*Posidonia oceanica*	Epiphytic	[100]
Antimicrobial activity	Epicotripeptin, Cyclo (L-Pro-L-Val), Cyclo (L-Pro-L-Ile), Cyclo (L-Pro-L-Phe) Cyclo (L-Pro-L-Tyr)	*Epicoccum nigrum*	*Thalassia hemprichii*	Endophytic	[101]
Promising antimicrobial activity (against *Staphylococcus aureus*, *Pseudomonas aeruginosa,* and *Candida albicans*)	Not identified	*Epicoccum nigrum*	*Thalassia hemprichii*	Endophytic	[102]
Moderate antibacterial activity against *Vibrio alginolyticus* and V. *parahaemolyticus*	Not isolated	*Aspergillus versicolor*	*Enhalus acoroides*	Not specified	[103]
Strong antibacterial activity against *Vibrio alginolyticus* and V. *parahaemolyticus*	Not isolated	*Aspergillus* *unguis*	*Thalassia hemprichii*	Not specified	[104]
A bacteriostatic effect (doctoral dissertation)	Not identified	Not given	*Thalassia testudinum*	Endophytic	[105]
Antiviral activity	Halovir	*Scytalidium* sp.	*Halodule wrightii*	Not given	[106]
Antiplasmodial activity (however, significant activity is not detected from seagrass fungi)	N/A	N/A	N/A	Endophytic	[107]
Cytotoxic activity against P388 and HeLa cancer cells.	Malformin A1	*Aspergillus tubingensis*	*Enhalus acoroides*	Not specified (should be endophytic)	[104,108]
In vitro cytotoxicity toward COLO 205 colon and SK-MEL-2 melanoma cancer cell lines.	Sansalvamide A	*Fusarium* sp.	*Halodule wrightii*	Surface fungi	[109]
Cytotoxicity against murine leukemia L1210 and human epidermoid carcinoma KB cells	Cladionol A (a new polyketide glycoside)	*Gliocladium* sp.	*Syringodium isoetifolium*	Not given	[110]
Cytotoxic activity against P388 and HeLa cancer cells	Not identified	*Penicillium thomii*	*Enhalus acoroides*	Endophytic	[111]
Anti-inflammatory activity	Thomimarine E	*Penicillium thomii*	*Zostera marina*		[112]
Production of hydrophobins (useful as biosurfactants)	N/A	*Penicillium chrysogenum*	*Posidonia oceanica*	Not specified	[113]
Production of lignin-modifying enzymes	Not isolated	*Flavodon flavus*	*Thalassia hemprichii*	Saprobic fungi (from leaves)	[114]
Lignocellulosic enzyme activities	Not isolated	*Flavodon flavus*	*Thallasodendon ciliatum*	Saprobic fungi (from decaying leaves)	[115]
Removal of polycyclic aromatic hydrocarbons (for bioremediation)	Not isolated	*Flavodon flavus*	*Thalassia hemprichii*	Saprobic fungi (from leaves)	[114]
Production of chitin modifying enzymes	Not isolated	Many fungal species	Many seagrass species	N/A	[63]
Production of xylan degrading enzymes, useful in biofuel industry	Not specifically identified	N/A	*Thalassia* sp. *Syringodium* sp. (active isolates are from these species)	Endophytic	[116]
Production of ligninolytic enzymes and tannases	Many species	N/A	*Posidonia oceanica*	Not specified (can be endophytic or epiphytic)	[73]
Decolorized bleach plant effluent from paper and pulp mill, a range of synthetic dyes and molasses	N/A	*Flavodon flavus*	*Thalassia hemprichii*	Saprobic fungi (from decaying leaves)	[117]
Isolation of new dimeric chromanone and a phthalide (the applications of them are not tested as they are in small quantities)	Bipolarinone, Bipolarilide	*Bipolaris* sp.	*Halophila ovalis*	Not specifically given (from leaves)	[96]
Isolation of a new β-resorcylic macrolide (5′-hydroxyzearalenone)	N/A	*Fusarium* sp.	*Thalassia hemprichii*	Leaves	[98]
Four new eudesmane-type sesquiterpenes thomimarines A–D (1–4)	Thomimarines A–D (1–4)	*Penicillium thomii*	*Zostra marina*	Superficial mycobiota of the rhizome	[111]
A new azaphilone derivative, xylariphilone	Xylariphilone	*Xylariales* sp.	*Halophila ovalis*	Not given	[118]
Weak to potent antimicrobial activity against the plant pathogenic fungi and bacteria	Isolated, but reference not available	*Aspergillus alabamensis*	*Enhalus acoroides*	Pathogenic (from necrotic leaves)	[119]
Weak to moderate antifungal activity towards phytopathogenic test fungi	Isolated	*Aspergillus insuetus*	N/A	N/A	[120]
Alleviate salinity stress in crop plants	Not isolated	*Trichoderma longibrachiatum*	*Posidonia oceanica*	Root endophytes	[121]

N/A = not applicable or not available.

## Data Availability

The original contributions presented in the study are included in the article/Supplementary Materials; further inquiries can be directed to the corresponding authors.

## Supplementary Materials

The following supporting information can be downloaded at: https://www.mdpi.com/article/10.3390/jof10090627/s1, Table S1: Previous records on endophytic and epiphytic fungi isolated from seagrasses using culture-dependent approaches. Table S2: Previous records on miscellaneous fungi isolated from seagrasses using culture-dependent approaches. Table S3: Previous records on pathogenic fungi-like organisms isolated from seagrasses using culture-dependent approaches. Table S4: Summary of the culture-independent studies on seagrass-associated fungi. References [15,35,47,57,59,60,61,62,64,65,66,67,68,69,70,71,72,78,80,82,83,84,92,93,101,102,105,118,119,133,134,135,138,144,145,190,191,192,193,194,195,196,197,198,199,200,201,202,203,204,205,206,207,208,209] are cited in the supplementary tables.
